# Accuracy of Guided Implant Surgery Using Tomographic Guides Combined with Functional Impression: An In Vitro Study

**DOI:** 10.34172/joddd.44401

**Published:** 2026-06-30

**Authors:** Renata Bommarito, Yolanda de Toledo Salvado da Ressurreição, Luciana Akemi Yamaguchi, Flavia Iotti Fernandes, Eduardo Perissinotto, Marcos Venturini Ferreira, Marcio Katsuyoshi Mukai, Roger Nishyama, Marcelo Munhóes Romano

**Affiliations:** ^1^Stomatology Department, University of São Paulo (USP), São Paulo, Brazil; ^2^Prosthodontic Department, University of São Paulo (USP), São Paulo, Brazil

**Keywords:** Dimensional measurement accuracy, Dental implant, Edentulous jaw, Functional impression, Guided implant surgery

## Abstract

**Introduction:**

Deviations between virtually planned and placed implants are still reported in guided implant surgery in completely edentulous arches. Associating a functional impression with the tomographic guide may enhance guide retention and reduce deviations. This study evaluated the effect of using a functional impression during tomographic image acquisition on the accuracy of implant placement in the edentulous maxilla.

**Methods:**

Fourteen 3D-printed edentulous maxillary models were fabricated. A complete denture was designed and printed for use as a tomographic guide for each model. During dual-scan image acquisition, the experimental group’s tomographic guides were seated with a light-body silicone functional impression, whereas the control group was scanned without impression material (n=7). After image registration in Blue Sky Plan software, implants were virtually planned, and surgical guides were printed for the placement of six implants per model. Postoperative scans were obtained, and STL files of planned and placed implants were superimposed to measure linear and angular deviations. Statistical analysis was performed using two-way ANOVA for functional impression and implant region (α=0.05).

**Results:**

No statistically significant differences were found regarding the effects of functional impression or implant position (*P*>0.05) on apical linear (2.11–3.25 mm), coronal linear (1.41–2.06 mm), or angular deviations (5.82–7.27°).

**Conclusion:**

The accuracy of implant positioning in edentulous maxillae was not significantly affected by the incorporation of a functional impression during tomographic scanning or by the implant region, although deviations were still observed. Further studies are necessary to evaluate the potential benefits of this approach in specific clinical scenarios.

## Graphical Abstract

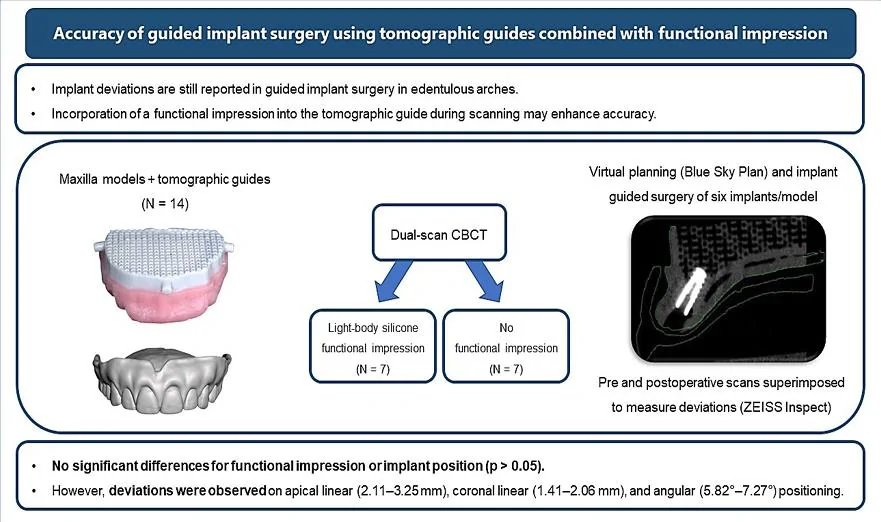

## Introduction

 One of the main challenges in complete-arch implant-supported prostheses is achieving precise three-dimensional positioning of multiple implants to support the prosthetic load with passive fit, allow effective hygiene, and ensure long-term esthetic and functional success.^1,2^ Virtual planning software and static guided implant surgery have been shown to improve the accuracy of implant placement in completely edentulous arches.^3-6^ However, deviations between virtually planned and actual implant positions are still reported.^3,7-11^

 The accuracy of guided implant surgery has been investigated in relation to several surgical variables, including flapless versus open-flap approaches,^11^ recent extractions,^12^ smoking habits and mucosal resilience,^8,13^ use of fixation screws,^11,13^ bone density,^8,14^ and differences between the maxilla and mandible.^3,8,13^ Yet, errors may also originate in earlier digital steps, accumulating throughout the workflow.^3,4,15-17^ One of the least-explored stages is image acquisition using the tomographic guide, which is typically the patient’s complete denture or a duplicate.^1^ The tomographic guide is associated with a hyperdense material that acts as a standard reference marker,^18^ enabling registration of images obtained in the dual scanning protocol within the implant planning software.^19^

 The tomographic guide must exhibit minimal distortion and intimate adaptation to soft tissues.^20^ In completely edentulous patients, mucosa-supported templates are inherently susceptible to displacement due to tissue resilience, which directly compromises the final transfer accuracy.^21^ Especially in resorbed ridges or flaccid mucosa, lack of guide stability or air gaps between the guide and mucosa can distort image registration and lead to errors during virtual implant planning,^18,22^ which may be subsequently transferred to the surgical guide. Incorporating a functional impression during the dual-scanning protocol may improve tomographic guide stability and image accuracy. Hyperdense materials such as polyether^23^ or certain light-body polyvinyl siloxanes^16,18^ can serve this purpose, acting simultaneously as a functional impression material and as a fiducial marker for the planning software.

 Therefore, this in vitro study aimed to assess whether incorporating a functional impression into the tomographic guide could improve implant placement accuracy in fully guided surgeries of edentulous maxillae. The null hypothesis was that the accuracy of implant positioning in edentulous maxillae would not be influenced by the functional impression during tomographic scanning or by the implant region.

## Methods

 The experimental design and digital workflow of this in vitro study are illustrated in Figure 1. A completely edentulous maxilla was digitally modeled using a software program (Meshmixer; Autodesk Inc, San Rafael, CA, USA), and geometric markers were added to the basal surface to facilitate subsequent image alignment. Fourteen maxillary models were printed with a digital light processing (DLP) printer (Straumann P20 + ; Institut Straumann AG, Basel, Switzerland) using a rigid resin (P Pro Model Gray; Institut Straumann AG, Basel, Switzerland) for the base and a 2-mm gingival layer (P Pro Gingiva Mask Pink; Institut Straumann AG, Basel, Switzerland) to simulate soft tissue (Figure 2). A complete denture was designed (Meshmixer; Autodesk Inc, San Rafael, CA, USA) to serve as the tomographic guide (Figure 3), and 14 tomographic guides were printed with the rigid resin (P Pro Model Gray; Institut Straumann AG, Basel, Switzerland).

**Figure 1:**
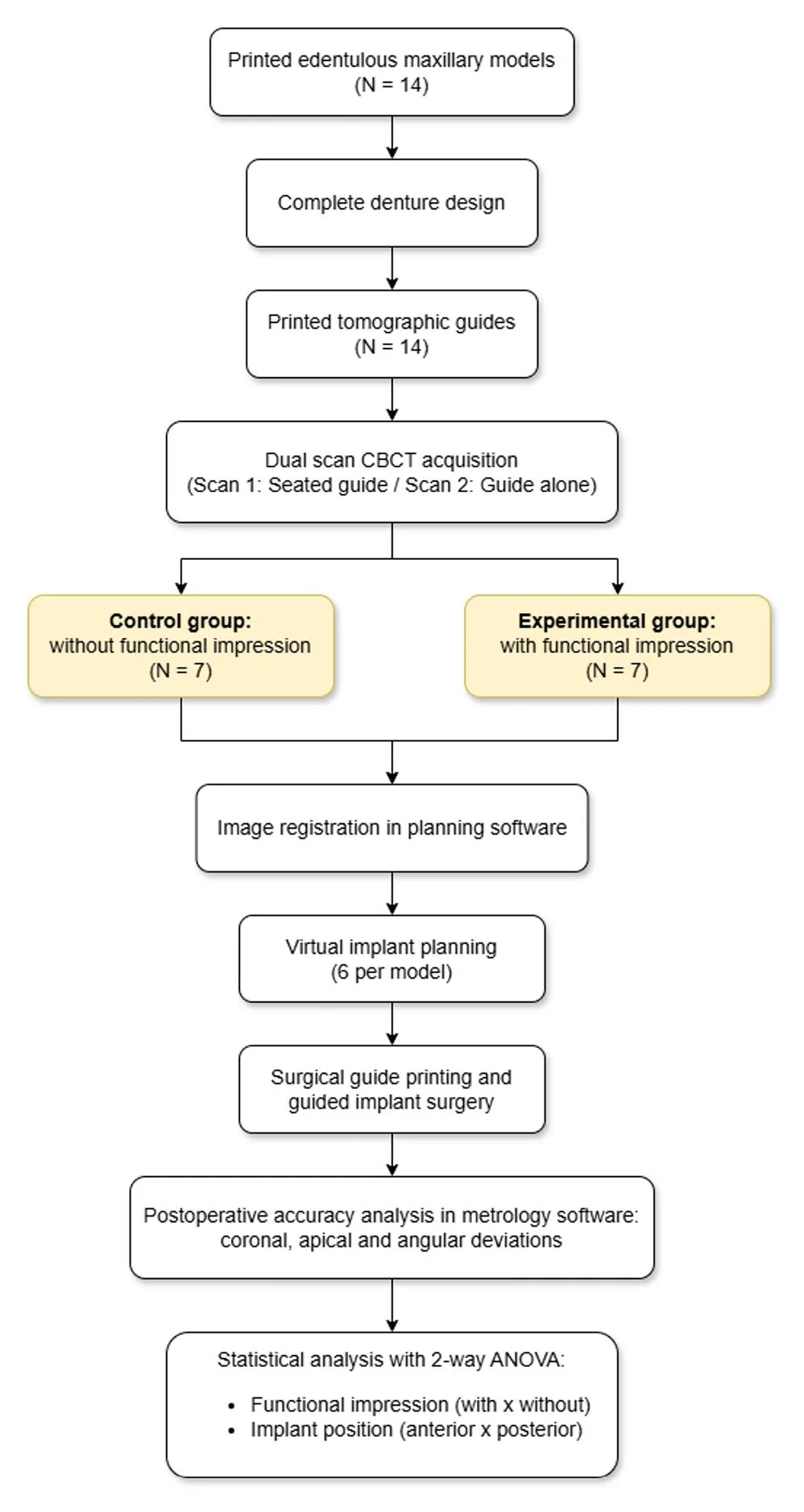
Flowchart representing the experimental workflow of the study

**Figure 2:**
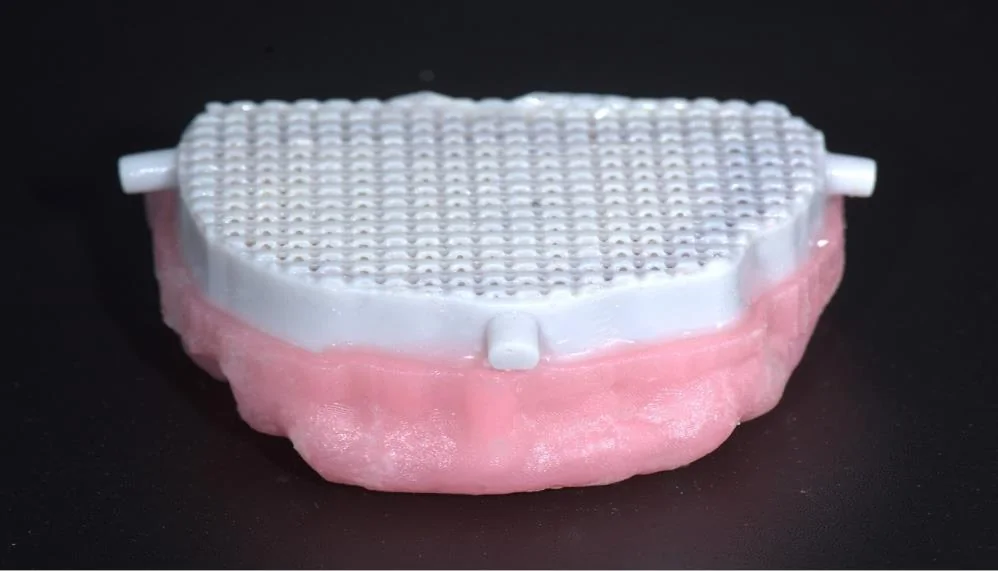
Maxillary model

**Figure 3:**
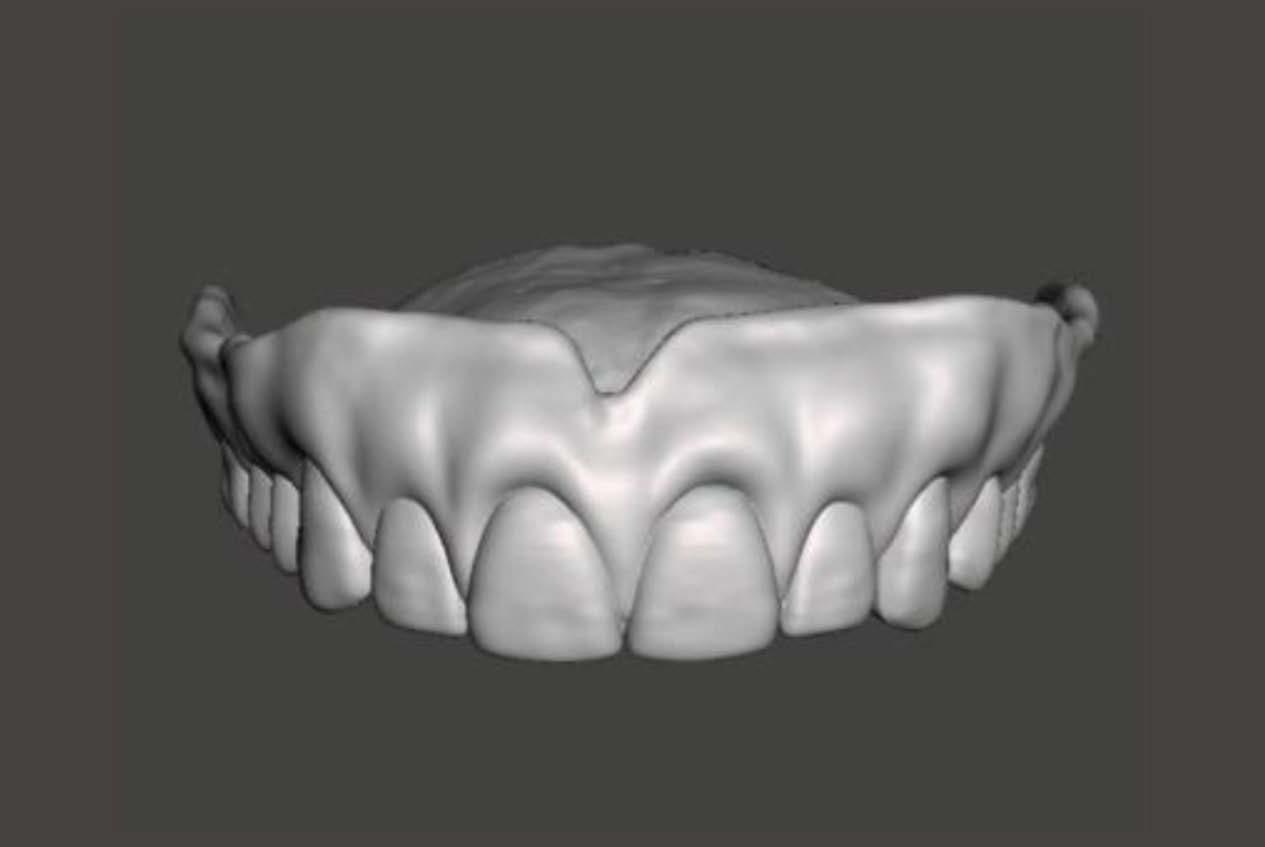
Tomographic guide design in Meshmixer

 Following the dual-scan protocol, two DICOM (Digital Imaging and Communications in Medicine) files were obtained for each model—one of the tomographic guide seated on the model and one of the guide alone—using a cone-beam computed tomography (CBCT) unit (Veraview X800; Morita Corp, Kyoto, Japan) at 70 kVp, 2 mA, an exposure time of 17.86 seconds, and standard resolution. The models and tomographic guides were allocated to groups by simple randomization using an online random number generator (Random.org; Randomness and Integrity Services Ltd, Dublin, Ireland). In the experimental group, tomographic guides were seated on the models using a light-body polyvinyl siloxane material (Panasil Initial Contact Light; Ultradent Products Inc, South Jordan, UT, USA) to simulate a functional impression along the intaglio surface and borders of the guide (n = 7), while the control group was scanned without impression material. Sample size was calculated in a statistical software program (G*Power; Universität Düsseldorf, Düsseldorf, Germany) using α = 0.05, β = 0.20, and a 1:1 allocation ratio for a two-tailed test, based on the mean and standard deviations obtained from a pilot study.

 The DICOM files were imported into the virtual planning software (BlueSkyPlan, version 4.12.13; Blue Sky Bio, Grayslake, IL, USA), and image registration was performed by automatically aligning the geometric markers on the model base, avoiding potential bias from operator or standard reference markers, which were not the focus of this study. Six implants (conical connection, 3.5 × 10 mm; Plenum® Bioengenharia, Jundiaí, SP, Brazil) were virtually planned in each model (Figure 4) at the sites corresponding to the central incisors, canines, and first molars, positioned parallel to each other and guided by the cingulum or central fossa of the tomographic guide teeth. Additionally, three fixation pins were planned: one in the midline and two between the canine and premolar regions. The files were exported back to the modeling software (Meshmixer; Autodesk Inc, San Rafael, CA, USA), and the internal portion of the model was trimmed to expose the planned implant geometry, which served as the reference image.

**Figure 4:**
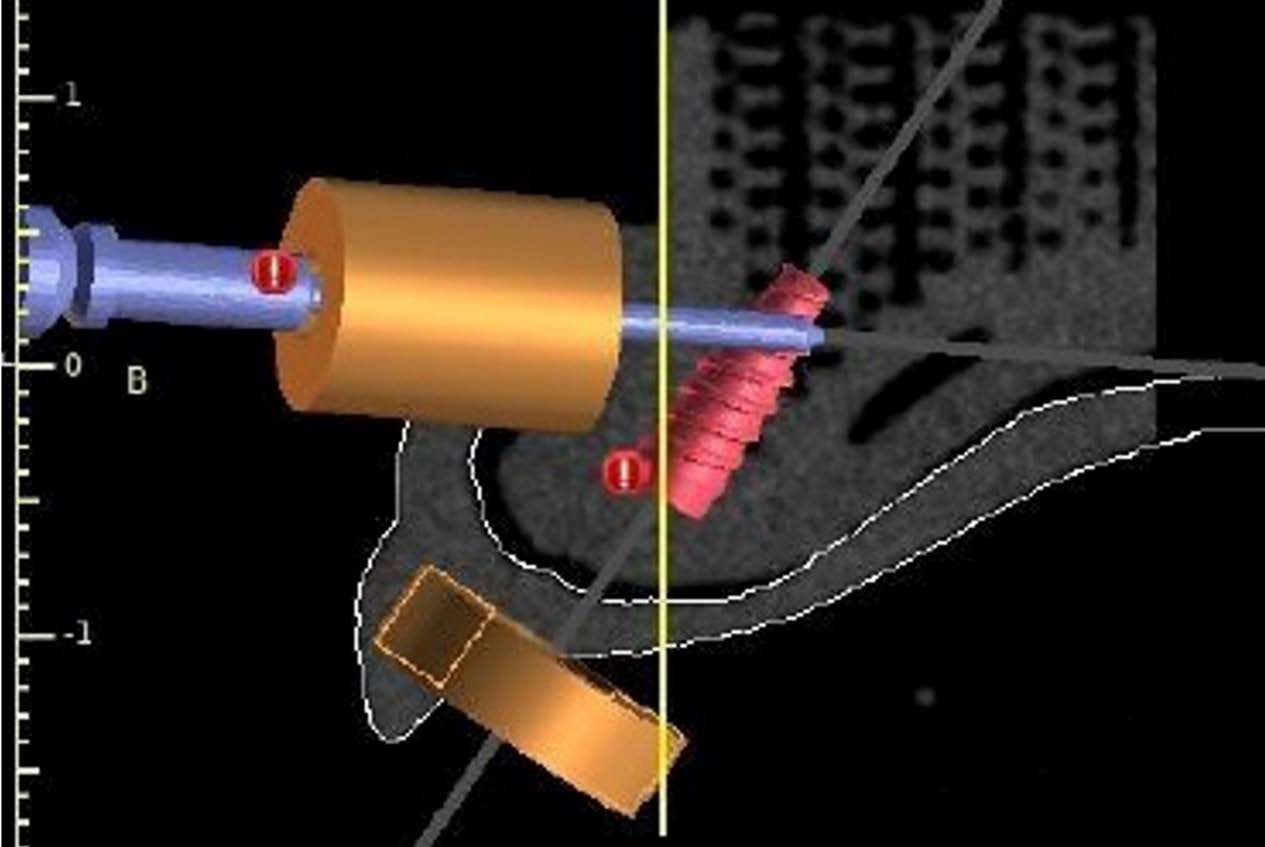
Sagittal view of the virtual implant planning in Blue Sky Plan. The white line represents the tomographic guide, gray denotes the maxillary model, red indicates the planned implant, and orange corresponds to the surgical guide design

 Following 3D printing of the surgical guides (P Pro Surgical Guide Clear; Institut Straumann AG, Basel, Switzerland), corresponding metal sleeves were inserted, and the templates were stabilized on each model with prefabricated fixation pins. Implant placement was performed by an experienced implantologist who was blinded to group allocation, using the specific guided-surgery instrumentation kit (Plenum® Bioengenharia, Jundiaí, SP, Brazil).

 Postoperative CBCT scans were subsequently obtained (Figure 5) and superimposed onto the virtual planning reference files using advanced metrology software (Zeiss INSPECT Optical 3D, version 2023.2.0.969, Zeiss Quality Suite; Carl Zeiss GOM Metrology GmbH, Braunschweig, Germany) via a surface-based best-fit alignment algorithm (Figure 6). Three-dimensional linear deviations were calculated as the distance between the central axes of the planned and placed implants at both the coronal and apical levels, whereas angular deviation was measured between their respective longitudinal axes. To ensure consistency and eliminate inter-examiner variability, all digital alignments and measurements were performed by a single, calibrated operator.

**Figure 5:**
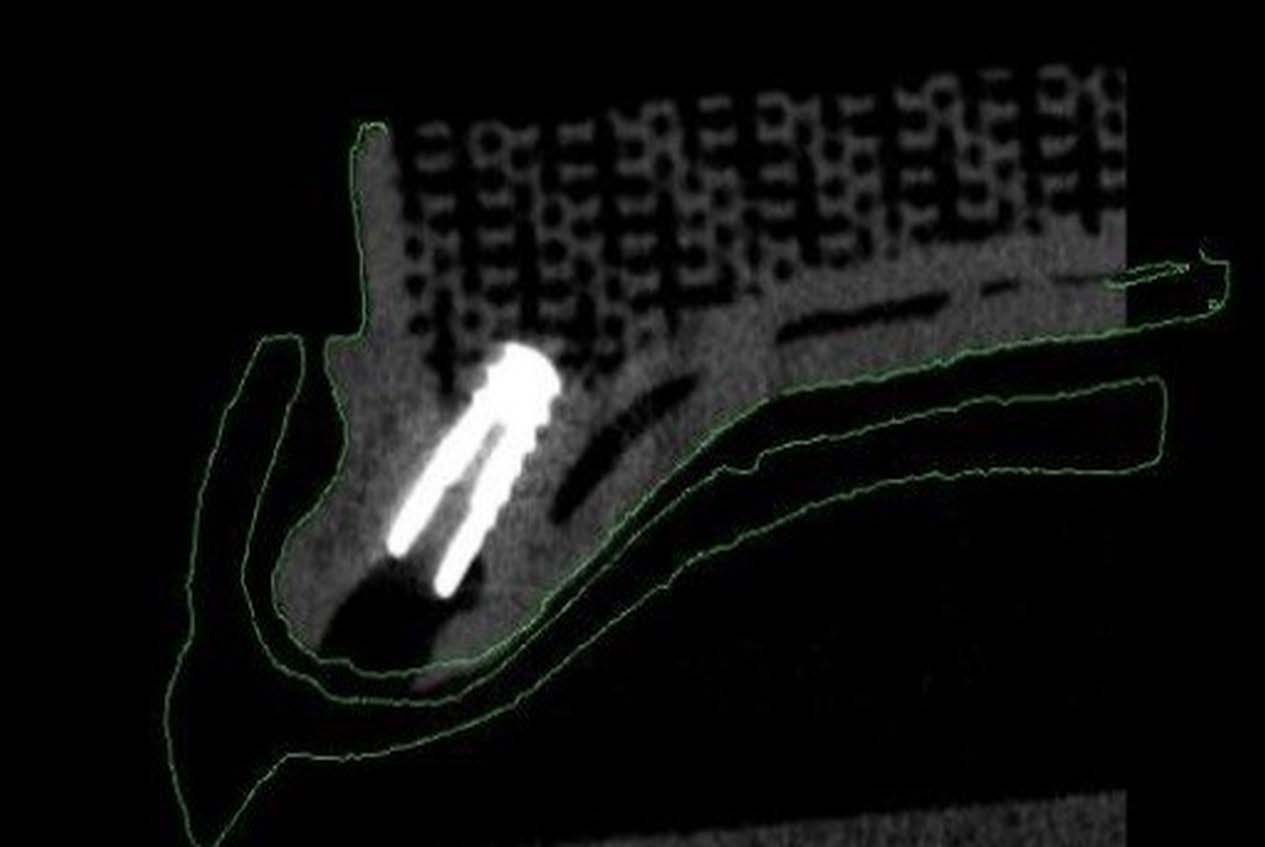
Sagittal view of the postoperative CBCT scan in Blue Sky Plan. Green represents the tomographic guide, gray denotes the maxillary model, and white indicates the placed implant

**Figure 6:**
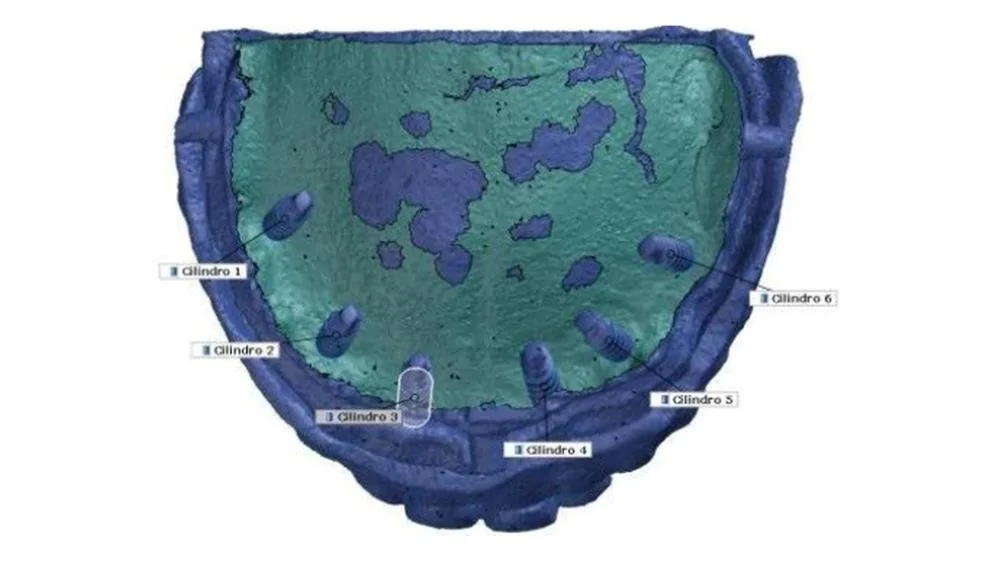
Implants and tomographic guide during image comparison performed in Zeiss INSPECT

 To account for the overall effect of the tomographic guide rather than individual implants, mean deviation values for the anterior and posterior implants were calculated for each model. Statistical analyses were performed using a statistical software program (JAMOVI version 2.3.13; The jamovi project, Sydney, Australia). A two-way analysis of variance (ANOVA) was conducted to evaluate the effects of functional impression (with or without) and implant position (anterior or posterior) on apical linear deviation, coronal linear deviation, and angular deviation (α = 0.05).

## Results

 Descriptive statistics for angular, apical linear, and coronal linear deviations are summarized in Table 1, and the results of the 2-way ANOVA are presented in Table 2. No statistically significant differences were found for the effects of functional impression, implant position, or their interaction on angular or linear deviations (*P* > 0.05).

**Table 1:** Means ± standard deviations for angular, apical linear, and coronal linear deviations according to functional impression (with or without) and implant position (anterior or posterior)

**Functional impression**	**With**		**Without**	
**Implant position**	**Anterior**	**Posterior**	**Anterior**	**Posterior**
Angular deviation (º)	6.777 ± 4.713	5.819 ± 3.528	5.967 ± 4.303	7.272 ± 2.923
Linear apical deviation (mm)	3.247 ± 0.909	2.641 ± 1.004	2.112 ± 0.396	2.432 ± 1.051
Linear coronal deviation (mm)	1.958 ± 0.374	2.059 ± 0.651	1.408 ± 0.353	1.952 ± 0.738

**Table 2:** Two-way ANOVA of functional impression and implant position for angular, apical linear, and coronal linear deviations

	**Angular deviation**		**Linear apical deviation**		**Linear coronal deviation**	
	**F**	***P***	**F**	***P***	**F**	***P***
Functional impression	0.04	0.843	3.496	0.076	2.097	0.163
Implant position	0.012	0.915	0.159	0.695	2.021	0.171
Functional impression X implant position	0.498	0.489	1.659	0.212	0.958	0.339

 Mean apical linear deviations ranged from 2.112 to 3.247 mm, coronal linear deviations from 1.408 to 2.059 mm, and angular deviations from 5.819° to 7.272°. Comparable angular values were observed for anterior (5.967–6.777°) and posterior (5.819–7.272°) implants. Although not statistically significant, posterior implants in the functional impression group exhibited slightly lower mean angular deviations (5.819°).

## Discussion

 The null hypothesis of this study was accepted, as the accuracy of implant positioning in edentulous maxillae was not significantly influenced by incorporating a functional impression during tomographic scanning or by the implant region. This outcome suggests that in controlled, stable in vitro settings, the additional step of a functional impression within the tomographic guide may not offer a cumulative accuracy benefit over a well-adapted guide.

 This lack of statistical significance can be largely attributed to the in vitro design. The standardized models used in this study had a uniform 2-mm simulated gingival layer with consistent rigidity and lacked the dynamic clinical variables often encountered in vivo, such as salivary flow, patient movement, and hyperplastic or highly resilient flaccid mucosa.^21^ In a clinical scenario, these mucosal variations may cause displacement or tipping of the tomographic guide during CBCT acquisition. Therefore, while a functional impression aims to compensate for tissue resilience and improve retention, its benefits might only become apparent when significant anatomical or tissue challenges are present, such as shallow vestibular anatomy, highly resorbed ridges, flaccid mucosa, and unstable prostheses.

 Despite the use of a fully guided surgical protocol with stabilization pins, noticeable deviations between the planned and placed implants were still observed across both groups. Mean coronal deviations reached up to 2.06 mm, apical deviations reached 3.25 mm, and angular deviations neared 7.27°. These values are slightly higher than those reported in recent systematic reviews of guided surgery in edentulous arches, which typically describe mean linear deviations under 2 mm and angular deviations below 5°.^3,9,10^

 Although a lack of standardized accuracy assessment methodologies limits meaningful comparisons across studies,^9^ the present findings highlight that other stages within the guided surgery workflow inevitably contribute to positional inaccuracies.^3,4,16^ Errors may occur as early as the DICOM data acquisition stage; previous studies have shown that the dual-scanning protocol introduces registration deviations depending on the type of fiducial marker^16,23^ or the imaging method employed,^16^ as well as potential tomographic artifacts that hamper image interpretation.^17^ To avoid potential bias from fiducial markers or operator point-based selection, image registration in the planning software was intentionally performed using the geometric markers on the model base for automatic alignment.

 Other surgical variables must be considered regarding their impact on implant placement accuracy. Cassetta et al.^15^ noted that the inherent mechanical tolerance (or “gap”) between the bur and the guide cylinder during surgical guide fabrication can lead to minor angular deviations in implant placement. Although fixation pins have been shown to improve implant positioning accuracy,^11,13^ Verhamme et al.^6^ and Tang et al.^5^ suggested that these pins alone may not be sufficient to completely eliminate template instability, due to micromovements caused by mucosal resilience or minor initial displacement of the guide from its planned position. Moreover, fixation pins are implemented exclusively during the surgical stage. Consequently, they cannot prevent potential errors originating earlier in the digital workflow—such as during tomographic image acquisition—from being transferred to the final virtual plan.

 The higher apical and angular deviations observed in the present study underscore the critical clinical need for a strict safety margin in virtual implant planning. Even under ideal in vitro conditions with a single, calibrated operator and a rigid model, deviations occurred. Therefore, a conservative safety zone of at least 2 mm away from vital anatomical structures—such as the maxillary sinus or nasal floor—remains mandatory, corroborating the guidelines proposed by previous authors.^3,10^

 Although computer-assisted implant workflows significantly improve placement accuracy, the cost of these treatments continues to limit their widespread use.^7^ Static guided implant surgery remains a reliable and predictable alternative for achieving accurate implant positioning, provided that its inherent limitations are carefully considered. If a functional impression does not actively improve final accuracy in standard edentulous scenarios, omitting this step could simplify the workflow, reduce chair time, and lower material costs without compromising the final surgical outcome.

 Nevertheless, the limitations of this study must be acknowledged. The rigid, non-dynamic nature of the 3D-printed maxillary models prevents direct extrapolation to clinical reality. Future clinical trials are required to determine whether the functional impression technique provides a stabilizing benefit for patients with anatomical conditions that severely compromise guide stability.

## Conclusion

 Within the limitations of this in vitro study, the accuracy of implant positioning in edentulous maxillae was not significantly affected by incorporating a functional impression during tomographic scanning or by the implant region, although deviations were still observed. Nevertheless, further studies are necessary to evaluate the potential benefits of this approach in specific clinical scenarios.

## Competing Interests

 There are no conflicts of interest to declare regarding this study.

## Ethical Approval

 Not applicable.
